# Motion of Balls, and Ball Wounds, Translated from the French of Legouest

**Published:** 1864-08

**Authors:** 


### CHRONICLE OF MEDICAL SCIENCE.

*Motion of Balls, and Ball Wounds.* Translated from the French of Legouest. (*Chirurg. Militaire.*)

*Motion of Balls.*—Projectiles are placed in motion by expansion of the gas from the powder; their form, their density, the arms from which they are discharged, the resistance which they meet with in the air, cause the motion which animates them to vary.

In military art, the *line of fire* is applied to the line of the axis of the piece indifferently prolonged, and which the projectile would follow if it was not subjected to the force of projection of the powder: the *line of aim* is a straight line passing over the most elevated point of the breech or the sight and muzzle of the piece: the *trajectory* is the curved line described in the air by the centre of the projectile; it departs from the line of fire at the muzzle of the piece where it is tangent to it, and remaining constantly below, it departs from it more and more as the projectile becomes more distant from the arm.

Balls are animated by a motion of translation according to the trajectory, and by a motion of rotation upon themselves. The direction of this motion, for spherical balls discharged from smooth-bore pieces, is left to chance; it is, on the contrary, communicated and calculated in advance, for oblong balls discharged from rifled arms.

*Motion of Spherical Balls.*—In smooth-bore arms, in order to facilitate the loading, there is left a certain difference between the cavity of the barrel and the calibre of the ball; this difference bears the name of *windage*. The result of this is, that when a round ball is placed in front of the powder, it rests generally upon the lower wall of the barrel, leaving a certain vacuum between itself and the upper wall of the barrel. Immediately upon ignition of the powder, a part of the gas product acts directly upon the ball, and causes it to take a motion forward; another portion of the gas escapes between the barrel and the ball, and exercises, in escaping, a certain pressure on the upper part of the latter. Obeying this pressure, the ball, instead of following a direction parallel to the axis of the barrel, strikes against the lower wall; then becoming elevated, it strikes against the upper wall; by successive shocks it gains the mouth of the piece and emerges, turning upon itself according to the direction of the last shock communicated to it. It happens that, by the intervention of the paper of the cartridge, the ball, instead of resting upon the lower wall of the barrel, may rest upon any point of the wall of the arm. Instead of vertical shocks, it may be subjected to shocks according to the diameter passing through its point of contact, and this varying indefinitely, it may, consequently, experience shocks directed according to every diameter of the barrel. If the last point of contact of the projectile with the arm, otherwise if the last shock took place on the inferior wall, the ball takes a motion of rotation from behind forward in a vertical plane, from above downwards; if it takes place on the upper wall, on the contrary, the ball takes a motion from below upwards; if it is the left wall which is last touched, the ball turns from right to left; if it is the right wall, the ball moves in the opposite sense; the same rule applies to all intermediate points.

This movement of rotation is not always concentric. When the centre of gravity does not correspond to the centre of the figure, it happens that the force of projection being applied to the mass, passes through the centre of gravity, while the resistance of the air, applied to the surface, passes through the centre of the figure; these two forces not being opposite, communicate to the projectile a movement of rotation the more eccentric as the two centres are more distant from each other.

The shocks and the indeterminate sense of the rotary motion of spherical balls discharged from smooth-bore arms, constituted elements destructive to the accuracy of the shot; therefore, various means have been tried for lessening them, through the medium of forcing the projectiles into the barrel. The forcing of the balls consists in diminishing, as much as possible, the windage of the arm—that is to say, the vacuum left between the interior walls and the projectile. This end was imperfectly attained, by surrounding the ball with a piece of buckskin or greased cloth; by flattening the ball more or less by repeated blows with the ramrod, and also by driving the rammer down with a mallet. The two latter modes of forcing, breaking the powder and destroying a part of its strength, were perfected by interposing between the ball and powder a circle of light wood, (*sabot*,) designed to bear the force of the blow and protect the powder; lastly, a chamber was fashioned in the breech, smaller than the anterior calibre of the barrel, destined to hold the powder, and on the orifice of which the ball rested and was battered down by the rammer.

*Motion of Oblong Balls.*—But these procedures, bringing with them new causes of deviation in the shot, were not slow in being abandoned, and were replaced by other means, founded upon the particular configuration given to the projectiles, (different oblong balls,) and the modifications applied to the interior wall of the arms, (rifled arms, chambered arms.)

All oblong balls have a motion helicoid to the axis of their translation.

They are forced down in various ways: In rifled chambered arms the cylindro-conic ball is driven with force by the rammer, the extremity of which is hollowed into a cavity corresponding with the point of the ball, in order not to deform it, the base of the ball reposes upon the margin of the chamber and sinks into it, to a certain extent, at each stroke of the rammer; the chamber prevents the powder being broken and reduced to dust. Compressed between the rammer and the chamber, the transverse diameter of the ball is augmented—it fills the calibre of the gun and takes the impressions of the grooved interior. The windage of the arm being thus done away with, the expansion of the gas, when the powder is ignited, acts entirely upon the base of the projectile; this escaping assumes a helicoid motion, directed according to the grooves.

The chamber opposing the cleansing of the arm, and becoming thus a cause of deterioration, was suppressed, and other means were advised for forcing the balls. The ball *à culot* was then brought into use; this ball is glided into the barrel and maintained in place by a few light strokes of the rammer; when the expansion of the gas takes place, the *culot* wedges into the hollow practiced in the axis of the ball, expands its walls and causes its external surface to penetrate into these grooves of the arm. It was soon perceived that the addition of the *culot* was useless, and that the same result was obtained with balls simply hollowed; these latter projectiles were substituted for the former, and are at this time in general use in the French infantry.

All balls, in their passage through the air, describe a curve, whose element changes at each instant of direction. In order that the oblong ball may maintain its point in front, an indispensable condition to the accuracy of the shot, it is necessary that the axis of the ball change in direction in the same manner as the element of the trajectory itself changes; and it is necessary that it be placed in the direction of the tangent to the trajectory. If it were otherwise, the projectile would assume a gyratory motion, and instead of arriving at its aim by the point, it might arrive at it by its base, by the transverse—in a word, by every point of its surface, like spherical balls.

It is to the action of the air that recourse is had for maintaining elongated balls in their normal direction. When an oblong ball passses from one element to the other of the trajectory, its axis is presented inclined to each new element of this line, but it is incessantly brought back in the direction of the tangent to the trajectory, by the increased resistence which opposes it, from the side to which its posterior part is inclined, the air taking effect in the channels practiced at the base of the projectile. Nevertheless, it is not always thus—in oblong balls not hollowed, the centre of gravity, situated behind the centre of the figure, is not constantly maintained in this position by the action of the air upon the channels, and tends incessantly to pass in front, causing the projectile often to see-saw and reach its aim by the base instead of its point.

Hollowed balls present less rarely this perturbation in their initial direction; the hollow in their base represents pretty nearly, in excavation, the full cone of their anterior part, and their centre of gravity is blended with their centre of figure, or even being placed a little in advance of it, has not the same tendency to become spontaneously displaced.

Beyond their motions of translation, rotation upon themselves (spherical balls,) and helicoid (oblong balls,) balls encounter other deviations, which are communicated to them by the resistance of the air, upon which they take their point of rest in turning. These deviations vary, for spherical balls, in the sense of their rotation upon themselves; for the oblong balls, animated by a regular helicoid motion, are always lateral, and are produced on the side opposed to that of the track of the helia.

*Velocity of Balls.*—Small projectiles, in general, are animated, at the moment of exit from the arm, with a very great velocity. The conditions of velocity depend very much on the quantity of powder composing the charge, the qantity of powder consumed at the moment of explosion, and the manner in which the weapon has been loaded.

The initial velocity of spherical balls from smooth-bore infantry muskets is sensibly greater than that of balls from rifled pieces. This difference is due to the resistance which forced balls encounter in rifled weapons, by their friction, their penetration or tearing in the internal ridges of the arm. A spherical ball fired from an infantry musket (pattern 1822) takes 0′′.42 to travel 150 mètres; an oblong ball, fired from a rifled piece, takes 0′′.50, to travel the same distance.

But the velocity of spherical balls decreases more rapidly than that of elongated balls; thus the spherical ball, which has at first traveled a distance of 150 mètres in 0′′.42, will take 1′′.75 to travel 400 mètres; while the elongated ball, which took 0′′.50 at the beginning of its course to travel 150 mètres, will only occupy 1′′·06 to travel 400 metres. It is at the distance of 200 mètres, about, that the comparative velocity of spherical and elongated balls begin to show a difference, which is in favor of the elongated ball, in proportion as the distance traveled increases. The conical form of the anterior portion of elongated balls, and their spiral motion upon themselves, diminishing the resistance opposed by the air to their progression, favors the prolonged conservation of their velocity.

*Penetrating Force of Balls.*—The penetrating force of balls is estimated by the number of boards they traverse; these boards, constructed of poplar wood and of the thickness of $0^{m}.026$, are placed at $0^{m}.50$ behind each other. The following tables show the difference in the penetrating force of round and elongated balls:

TABLE I.

| | | | BOARDS. | | | |
|---|---|---|---|---|---|---|
| | | | No. 1. | No. 2. | No. 3. | No. 4. |
| Spherical balls fired from smooth-bore muskets, at distance of 400 mètres, | 120 | Traversed, | 2 | 1 | 0 | 0 |
| | | Lodged, | 2 | 0 | 0 | 0 |
| | | Made impres'n, | 0 | 1 | 0 | 0 |

Six balls out of 120 reached the mark; only one traversed the first and second boards; one other made impression upon the second board after traversing the first; two traversed the first board only; two others only lodged in the first.

TABLE II.

| | | | BOARDS. | | | | | | | |
|---|---|---|---|---|---|---|---|---|---|---|
| | | | No. 1. | No. 2. | No. 3. | No. 4. | No. 5. | No. 6. | No. 7. | No. 8. |
| Oblong balls fired from rifled muskets, at the distance of 400 mèt's, | 120 | Traversed, | 63 | 53 | 52 | 43 | 32 | 14 | 3 | 1 |
| | | Lodged, | 00 | 3 | 10 | 2 | 4 | 1 | 0 | 0 |
| | | Made impres'n, | 00 | 5 | 3 | 6 | 7 | 12 | 7 | 0 |

The figures of Table No. 2 prove, without its being necessary to compare them with Table No. 1, that the accuracy and penetrating force of elongated balls are considerably greater than those of spherical balls. Physics teach us that the motive quantity of bodies is equal to the product of their weight by their velocity; according to this principle, elongated balls being much heavier than spherical, possess a motive quantity superior to the latter, notwithstanding they have an inferior velocity at their exit from the weapon. The resistance of the air then takes longer to overcome the motive quantity of oblong than of spherical balls. The velocity of the new decrease, necessarily, more slowly than of the ancient projectiles; thus it is explained how the former may, at first, be surpassed in velocity by the latter, then progressively acquire an equal, and lastly a superior, velocity.

It is then necessary, whenever we wish to compare the effects of spherical balls with those of elongated, to estimate accurately the distance at which they have been fired.

Balls fired from smooth-bore weapons undergo no deformity before their exit from the weapon; forced balls, on the contrary, are subjected to those deformities which may be called normal. The shock of the rammer, the action of the chamber upon the base of the ball, the impression of the grooves upon its surface, the enlargement or tearing of its cylindrical portion, are so many causes of constant deformation which we meet with, and which vary their aspect according to the mode of forcing employed.

### *Wounds from Balls.*

*Penetration.*—When a projectile strikes the parts of an angle more or less approaching to a right angle, it may make only one opening, and, in this case, it is quite likely it remains in the tissues in a sort of cul-de-sac; or else it may make two openings, the one of *entrance*, the other of *exit*.

*Openings of Entrance and Exit.*—The two openings made by the ball, that of entrance and that of exit, have not always such distinct characters as to be distinguished from each other; their relative size has served as a test for long discussions, in which surgeons equally expert have given opinions diametrically opposite. Some contend to have always seen the wound of entrance larger than that of exit; others pretend to have always seen the contrary, and contend that the opening of entrance was larger than that of exit.

The wounds of entrance and exit are sometimes equal, sometimes unequal in their size; this surpassing that, and reciprocally, as Huguier has proved in his researches touching this subject.

Equality in the wound of entrance and that of exit is only possible when the ball, at its entrance, strikes the skin under an incidence equal to that which it possesses in traversing it for its exit; when the impulse of the ball has not sensibly diminished during its passage through the parts; when the tissues traversed are of equal density; when the skin on the side of the entrance wound possesses the same laxity, the same elasticity, and the same thickness as on the side of the exit wound; lastly, when the ball is not deformed, whether in its course through the free air, whether in its passage through the economy, and presents itself to the tissues and the integuments always in the same position.

The opening of entrance is smaller than the opening of exit, when the ball encounters, in the measure of its advance, more dense tissues, as the debris of aponeuroses, of tendons or bones, dragged along in its course; when it becomes deformed in its passage through the thickness of the parts themselves, when, deformed in its aerian passage, it penetrates in presenting a surface of less diameter than that which it presents at its exit; when the skin glides upon the aponeuroses or upon the bones; lastly, when the ball enters perpendicularly to the parts, and makes its exit in a direction oblique to their plane.

The opening of entrance is larger than the opening of exit, when the ball penetrates obliquely and makes its exit perpendicularly to the parts; when the ball is fired at a short distance; when the projectile carries with it the wadding or portions of the garments, and which it leaves in the parts; when the living tissues are more dense on the side of entrance than that of exit; when the skin glides over aponeuroses or over bones; lastly, when the ball, at its entrance, presents itself under a larger surface than at its exit.

The comparison established by Dupuytren between the physical effects of projectiles impelled by gunpowder upon inert bodies, and their effects uppon living bodies; the experiments made, under his direction, by Paillard, and repeated in 1848 by Huguier, cannot be accepted in an absolute manner, and are only applicable in what concerns the solid or osseous parts of the skeleton. Clinical examination, on the one part, and, on the other, experiments upon living animals and upon the dead, are the only sources from which we should draw, for the effects of balls, notions which a long practice may be said to multiply to infinity.

The wound of entrance of balls pretty generally represents the form of the projectile. Round balls most often make a rounded opening of entrance, the dark borders of which are depressed and inverted; the external circumference of the wound is almost always pretty clearly defined by an ecchymotic border; the internal circumference irregularly defined by contusedor disorganized borders; its centre presents, sometimes, a loss of substance; at other times, it is occupied by shreds of dermis and mortified cellular tissue. Blood in small quantity or brown-colored sanious liquid flows or oozes from the interior of the wound; sometimes there is no discharge of any character from it.

The dark color of the wound is due in part to the contusion, and in part to the abandonment, by the projectile, of the sediments of the powder, or the gaseous products of the powder, with which it had been in contact for some moments.

The wounds of entrance, and sometimes, but much more rarely, the wounds of exit, exhale an odor manifestly sulphurous, due, like the dark color, to the presence and to the solution of deposits of powder ignited upon the projectile.

The sulphurous odor of wounds must not be confounded with the odor brought by the wounded from the battle-field, whose hands and faces are often blackened by powder, after repeated usage of their arms.

These different phenomena, and in particular the aspect of gunshot wounds, made the older surgeons, who observed them, believe that the projectiles were poisonous, or burned the tissues through which they passed. The latter error reigned until 1564, when A. Paré combatted it victoriously, and showed that *if a ball was fired into a sack of gunpowder it was not ignited.* It is probable, however, that the temperature of projectiles is elevated a little, since the arms gradually become warmer after each discharge, to such an extent that the fire has to be intermitted to avoid accident; but the arms, otherwise made of metals better conductors of caloric than of lead, to the influence of which the projectiles are only subjected for an instant, and which cannot communicate to them a temperature sufficient to burn, and much less to cauterize the parts through which they pass. As to the poisoning of wounds, it has no more existence than the burning; but the decomposition of the organic strata bruised by the projectile may give rise to dangerous compounds, when they pass into the torrent of the circulation, and thus considered, gives a satisfactory solution of the opinions of ancient surgeons upon gun-shot wounds.

The wounds of exit of spherical balls are generally more irregular than those of entrance; they often present lacerations, and sometimes shreds disposed in rags; the skin seems to have bursted; the borders, much less contused than those of entrance wounds, are habitually more or less everted. The peripheric ecchymosis sometimes does not exist the first day of the wound; it generally does not appear for several days, and is of considerable extent.

*Multiple Wounds.*—Instead of making one only or two wounds, constituted by the openings of entrance and exit, balls may make multiple wounds, and it is not rare to see three, four, five, and even more wounds produced by the same ball, which in its track has en-encountered successively many parts of the body. Six wounds have been recognized, in one soldier, made by the same ball having traversed both thighs and the scrotum.

On the other hand, two balls arriving together or at close intervals, the wounded having maintained the same position, may enter by a single opening and remain in the parts, or may make their exit by two separate openings.

The details into which we have entered, give an idea of the general aspect of wounds produced by projectiles from fire-arms, and of their action upon the skin. Some surgeons have believed that the openings made by oblong balls were not so lacerated as those made by spherical, when the discharge was in close proximity; that they were oblong, well defined, sometimes linear, smaller than those of exit; that the openings of exit were more regular, longer than broad, if the ball followed its primitive direction; irregular, very much lacerated, with edges considerably elevated, if the ball deviated and made its exit transversely. But our own observations have convinced us that, if the openings of entrance and exit of oblong balls present a variety of aspect and of form greater than the openings of spherical balls, they do not differ essentially, and that their dimensions are equally subjected to the conditions which we have heretofore exposed. Cases have been cited where oblong balls have made linear openings, but analogous phenomena have been observed in the effect of spherical balls. These phenomena ought to be regarded as very rare and quite exceptional; they are produced under conditions, as yet, poorly defined, and seem to us to reside in the laxity and the folds of the skin struck in the side of flexion of the articulations, when these are strongly flexed.

*Track of Balls.*—The question of knowing how projectiles penetrate the parts—that is to say, if they penetrate by pushing aside the tissues, by dividing them or by destroying them—is an idle one, inasmuch as they comprehend all these different modes of action, modified by the force which animates them and which makes them contusing bodies. Oblong balls, themselves, in spite of their conical anterior, in spite of the gimlet-motion which is communicated to them, do not act otherwise. Some fortuitous circumstances, impossible to foresee and difficult to explain, may, without doubt, cause a ball to penetrate in one way rather than another; but, in the immense majority of cases, the mode of penetration is such as we have indicated.

Whatever may be the case, when a ball penetrates to a certain depth or traverses a certain part of the body, it encounters in its track tissues and organs of different natures, upon which it acts in different manners. The cellular tissue, distributed in such abundance throughout the whole economy, is always perforated; according to the elasticity with which it is endowed, it presents a canal of greater or less diameter, the walls of which are more or less contused, while the centre corresponding to the axis of the track of the projectile is destroyed or mortified. The rectitude, the regularity and the width of the canal depend still upon the volume, the form and velocity of the projectile upon the tension or relaxation of the parts struck.

The aponeuroses and the fibrous tissues, in general, may suffer a loss of substance; but oftenest they only present lacerations more or less irregular: the tissues sometimes separate, allowing the projectile to pass through a simple cleft in the direction of the aponeurotic or ligamentous fibres, and which, approaching, dissemble the track of the ball. Solid aponeuroses may resist the action of projectiles and remain intact in appearance, while the subjacent tissues are more or less contused. Tendons, in general mobile, always lubricated by the synovial fluid, gliding easily upon one another, may escape the action of balls, but may also be lacerated or contused; their fibres being dissociated or destroyed.

The muscular tissue yields readily to the action of projectiles which divide it, tear it and traverse it, making furrows and canals, with walls more or less regular, and tracks more or less direct. These phenomena depend upon the incidence according to which the muscles have been struck by the projectiles, and of the state of tension, of contraction or relaxation in which they have been surprised. The muscles are equally traversed by balls when they are stretched, contracted or relaxed: it is impossible to say in which of these states they yield to or resist the action of projectiles. Tension and contraction ought to seem to favor the division of muscles, by offering to the balls a certain degree of resistance necessary to insure their action; but they may also determine deviations. Relaxation, it appears, ought to lessen the shock of the ball and allow of the slipping of the muscle, which enables it to escape the projectile. The tense muscular fibre offers less surface to the effect of balls than the reverse; the result is, that perforation or loss of substance in a tense muscle becomes less apparent when the muscle is placed in a relaxed position; the contrary is observed in a case where the muscle has been divided or perforated during its contraction. These conditions, joined to the simultaneous lesion of many muscles which, according to the movement executed by the wounded, are the ones tense, the others contracted or relaxed, and which, in a state of repose no longer preserving the same relation with one another as during their action, cause sinuosities in the track of the ball, and may interrupt its continuity.

All the other soft tissues of the economy yield to the effects of balls; lacerated, divided, perforated or disorganized, they comport with the anatomical texture with which they are endowed. The ones, fixed, cannot escape the direct or oblique action of balls; the others, mobile, sometimes avoid them.

The hard parts, that is to say, the cartilages and the bones, according as they are struck perpendicularly or directly by the balls, suffer different lesions: the cartilages, by their suppleness and elasticity, sometimes escape the action of projectiles; but they may be also contused, fractured or destroyed: the bones, by reason of their density, may resist projectiles, but they are also contused, perforated, split, fractured and disorganized.

These different considerations are so many elements proper for a general appreciation of the form of the track of balls. Dupuytren, who thought that a ball fired at a certain distance always made a wound of entrance smaller than that of exit, also admitted that the ball made a canal broader in proportion as it advanced in the parts. For him, the track of a ball is represented by a cone, the apex of which corresponds to the wound of entrance, and the base to the wound of exit. Experience does not conform to this view of the matter. When the finger is introduced into the track of a ball, it penetrates without difficulty: contracted at the opening of the skin and superficial aponeurosis, it moves beyond, in effect, in a freer space; but, if it meets more deeply with new aponeuroses, tendons, nerves or vessels, ligaments or bones, it encounters either an obstacle or a new constriction, and recognizes numerous irregularities in the route over which it passes. The track of a ball, however direct it may be, does not then constitute a canal of equal calibre throughout its whole extent, or more considerable according as it is more deep; it presents, on the contrary, irregular walls, sometimes approximated, sometimes removed from each other, according as it involves tissues which have suffered, each one after its manner, the action of the projectile. Certain ones have only been torn, others have been destroyed; these have returned upon themselves—those, on the contrary, have become separated; the ones, struck with immediate death, have remained in place, the others have been carried away; all have been violently contused.

*Deviation of Balls.*—Spherical balls do not always make a rectilinear track in the parts wounded or traversed; they deviate sufficiently often from their original direction, sometimes to take such a singular one, that it is difficult if not impossible to give a satisfactory explanation for it. The variable incidence of projectiles upon parts, the difference of density presented by these; the deformation of the projectiles themselves and the motions which animate them; the encounter of aponeuroses more or less tense over which they may glide, of tendons, of muscles more or less contracted, of cartilages endowed with certain elasticity; the shock against bones fractured or remaining entire, are causes which make balls deviate from the direction which they possess at their moment of penetration.

The greater portion of authors who have written upon wounds by fire-arms, cite a number of singular deviations of projectiles. The most remarkable deviation that we have seen, among a very large number, is that of a Biscaian, which, penetrating near the left angle of the inferior maxillary, stopped under the skin between the last false rib and the ilium of the same side: the soldier was struck (battle of Inkerman) while standing, and fell immediately. It results from these facts that projectiles are sometimes arrested under the skin, or make their exit at considerable distances from where they penetrated, lodging or escaping at points entirely unforeseen.

According to some surgeons—Scrive, Quesnoy, McLeod—oblong balls have in general a more direct track than round balls: but we, as well as our confreres in the late campaigns, have seen that they also vary very often. The cone composing their anterior part presents surfaces perfectly disposed to cause deviations of projectiles, when, instead of striking the resisting parts directly by the point, they come in contact by a curved or oblique portion of their surface.

Spherical balls striking obliquely upon the walls of a cavity or the circumference of an articulation, have sometimes circumscribed the cavity or articulation without penetrating, and have made their exit, or presented themselves under the skin, at a point more or less distant from their entrance, sometimes even at a point diametrically opposite. We have seen balls traverse a limb from part to part without fracturing the bones, notwithstanding these found themselves directly in its track. Others, but more rarely, have penetrated into cavities without injuring their organs, notwithstanding their lesion seemed inevitable.

Levacher explained this phenomenon, by saying that the skin or the external wall of the track of the ball resists laterally; if the resistance is greater than the effort of the ball, this, without making its exit, will pass on to a more distant point, when, still encountering a greater resistance, it will progress anew until it has lost all its motion, or until the skin presents itself in such a manner that it can act with all its force.

Dupuytren has accepted and repeated this theory, which gives the fact without explaining it.

We readily admit that solid or elastic walls, like those of the vault of the cranium and of the chest, when they are struck obliquely in their concavity, resist, in a certain measure, the action of a ball, but we cannot admit that the skin alone can act in the same manner, especially when the projectile is animated with a force sufficient to travel over the half or even the fourth of the circumference of the integuments of the abdomen, the cranium or the chest. We do not think that balls can circumscribe the walls of a cavity, under the integument, unless they are animated by a motion of rotation upon themselves, which they execute in a determined sense. According to our idea the thing occurs thus: When a ball strikes obliquely upon the sternum or a rib, and traverses the skin, it is animated, besides its movement of translation, the direction of which is changed by the resistance and elasticity of the bone, by a movement of rotation upon itself. If this movement of rotation is executed in such a way as to make the projectile turn from behind forwards, and allows it to roll, so to speak, upon the internal surface of the integuments, the projectile will be able to travel over a circular track until its rotary motion is expended. As soon as it no longer exerts this movement, it will obey that of translation and will perforate the integumentary wall. If, on the contrary, the rotatory motion of the ball is from before backwards, in such a way that it cannot roll in the sense of its translation, on the internal surface of the skin, and that it pushes before it the tissues acting as obstacles, it will soon overcome the resistance and make its exit, after having made a very short track, in the direction which the shock upon the bone has communicated to it.

It is necessary, also, in order that a ball circumscribe a cavity, that it shall not be deformed, and that its surface, perfectly smooth, present no asperity capable of tearing the internal surface of the skin.

The considerations into which we have entered make it plain that oblong balls cannot travel similar routes. Observation is here in keeping with theory. Oblong balls, of more or less irregular configuration and animated by a spiral movement, have offered no example of these deviations, commonly enough observed in the track of spherical balls, to have been signalized by the majority of surgeons.

To achieve our general aim, the parallel which we have sought to establish between spherical and oblong balls, we will say that the greater accuracy in the discharge of the latter seems to give a larger number of wounded in modern wars. Colonel Nilford, instructor at the English School of Practice, reports that in Cafraria 80,000 round balls were fired from old muskets and wounded 25 Caffres; while at Cawnpore, one company armed with English carbines, attacked by a squadron of cavalry, dismounted 69 assailants at one discharge. It has been calculated that at Salamanca, out of every 3,000 balls fired by the English army, only one took effect. At Solferino, (1859,) in twenty-four hours, 11,500 French, 5,300 Sardinians and 21,000 Austrians were placed *hors de combat;* while the reports of the English army prove that, during the battles of the 16th, 17th and 18th of June, 1815, including the battle of Quatre-Bras and that of Waterloo, the wounded numbered 8,000, without counting those of the Duke of Wellington.

These considerations have a great importance with reference to the organization of the surgical assistance required by the armies of our days.

